# Human Epidermal Growth Factor Receptor 2 (HER2/Neu)-Positive Ovarian High-Grade Serous Carcinoma Metastasizing to the Breast: A Case Report

**DOI:** 10.7759/cureus.92910

**Published:** 2025-09-22

**Authors:** Edoardo G Frezza, Gayatri D Nimmagadda

**Affiliations:** 1 College of Medicine, Trinity School of Medicine, Baltimore, USA; 2 Hematology and Oncology, University of Maryland Baltimore Washington Medical Center, Glen Burnie, USA

**Keywords:** breast metastasis, her2-positive breast cancer, her2-positive ovarian cancer, metastatic ovarian carcinoma, mullerian origin breast cancer, ovarian high-grade

## Abstract

Metastasis to the breast from malignancies is rare and often poses a diagnostic challenge due to its clinical and radiologic similarity to primary breast cancer. We report the case of a 47-year-old woman who presented with abdominal discomfort, a palpable abdominal mass, and bilateral inguinal lymphadenopathy. Imaging and biopsy revealed high-grade serous carcinoma of Müllerian origin with human epidermal growth factor receptor 2 (HER2/neu) overexpression, which had metastasized to the breast and right axillary lymph nodes. She was treated with a combination chemotherapy regimen of Taxotere, carboplatin, trastuzumab, and pertuzumab (TCHP). This case underscores the importance of distinguishing metastatic involvement of the breast from primary breast cancer to ensure appropriate treatment and management.

## Introduction

Metastatic disease to the breast from malignancies is an uncommon occurrence, often presenting a diagnostic challenge due to its resemblance to primary breast cancer. Among the various malignancies capable of spreading to the breast, ovarian carcinoma is an infrequent source, making such cases particularly noteworthy. High-grade serous carcinoma, the most prevalent and aggressive form of ovarian cancer, primarily spreads within the abdominal cavity, with distant metastases occurring less frequently. When these metastases do involve the breast, they can mimic primary breast cancer both clinically and histologically, necessitating careful evaluation to ensure accurate diagnosis and appropriate management. Additionally, metastasis is typically triple-negative (ER, PR, and human epidermal growth factor receptor 2 (HER2/neu) negative). This case report discusses a rare presentation of metastatic high-grade serous carcinoma of Müllerian origin with HER2/neu positivity, highlighting the importance of immunohistochemical analysis and a multidisciplinary approach in guiding treatment decisions.

## Case presentation

Our patient is a 47-year-old woman who presented with abdominal discomfort. On physical exam, there was a large palpable abdominal midline mass and bilateral inguinal lymphadenopathy. Complete metabolic panel and hematological indices were unremarkable. Patient's CA-125 levels were elevated at 988 U/mL. She was negative for the BRCA1/2 gene mutation. Advanced tumor analysis reported HER2/neu positive, homologous recombination deficiency (HRD) negative, Tp53 positive, PDL-1 positive, ARID1A positive gene mutations.

**Figure 1:**
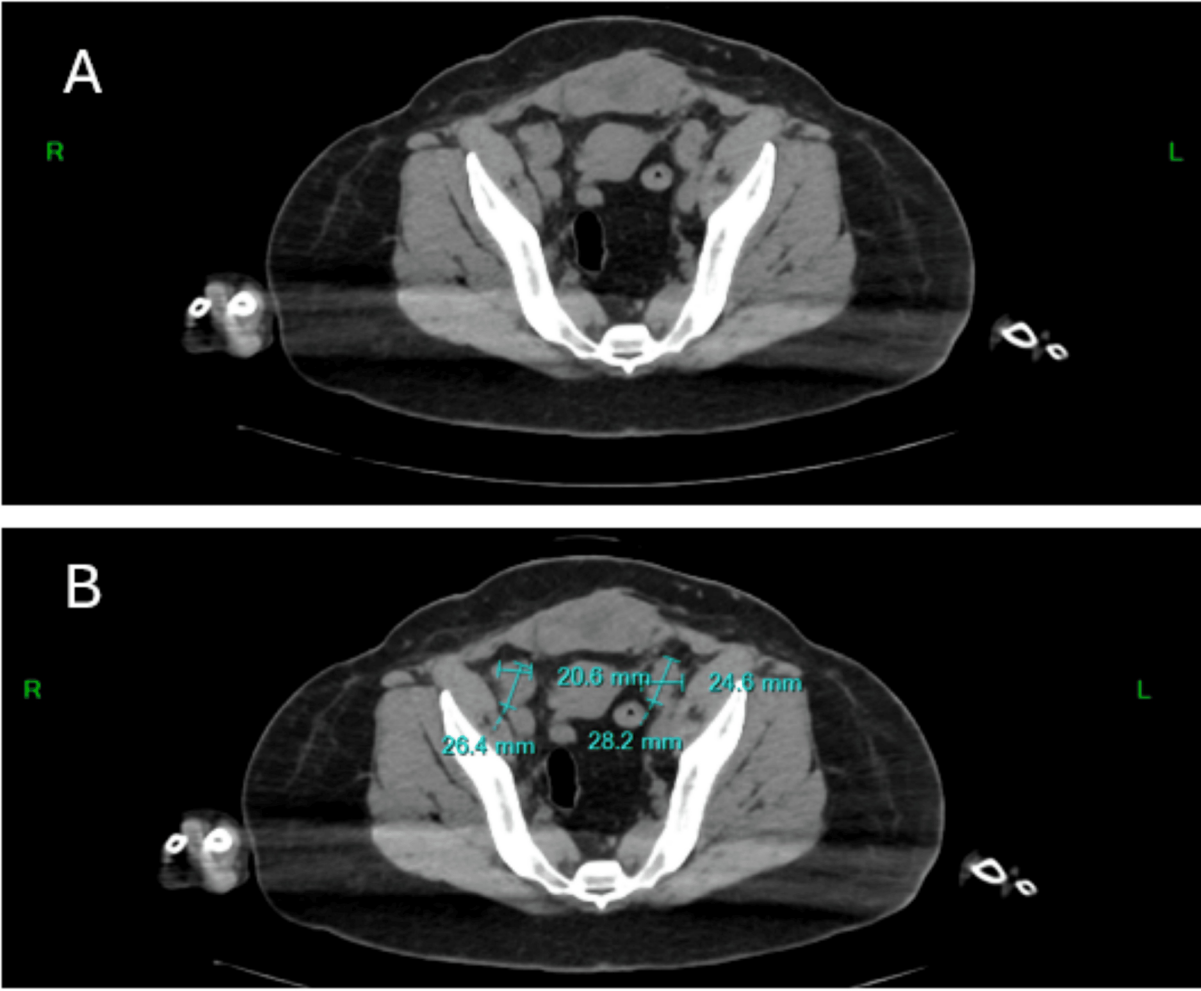
CT abdomen and pelvis. Axial slice of the patient showing the identified masses within the pelvis. (A) The patient's uterus is shown in the top portion of the scan with two masses on either side of it. (B) The measurements are superimposed for identification of the masses on either side of the uterus.

**Figure 2:**
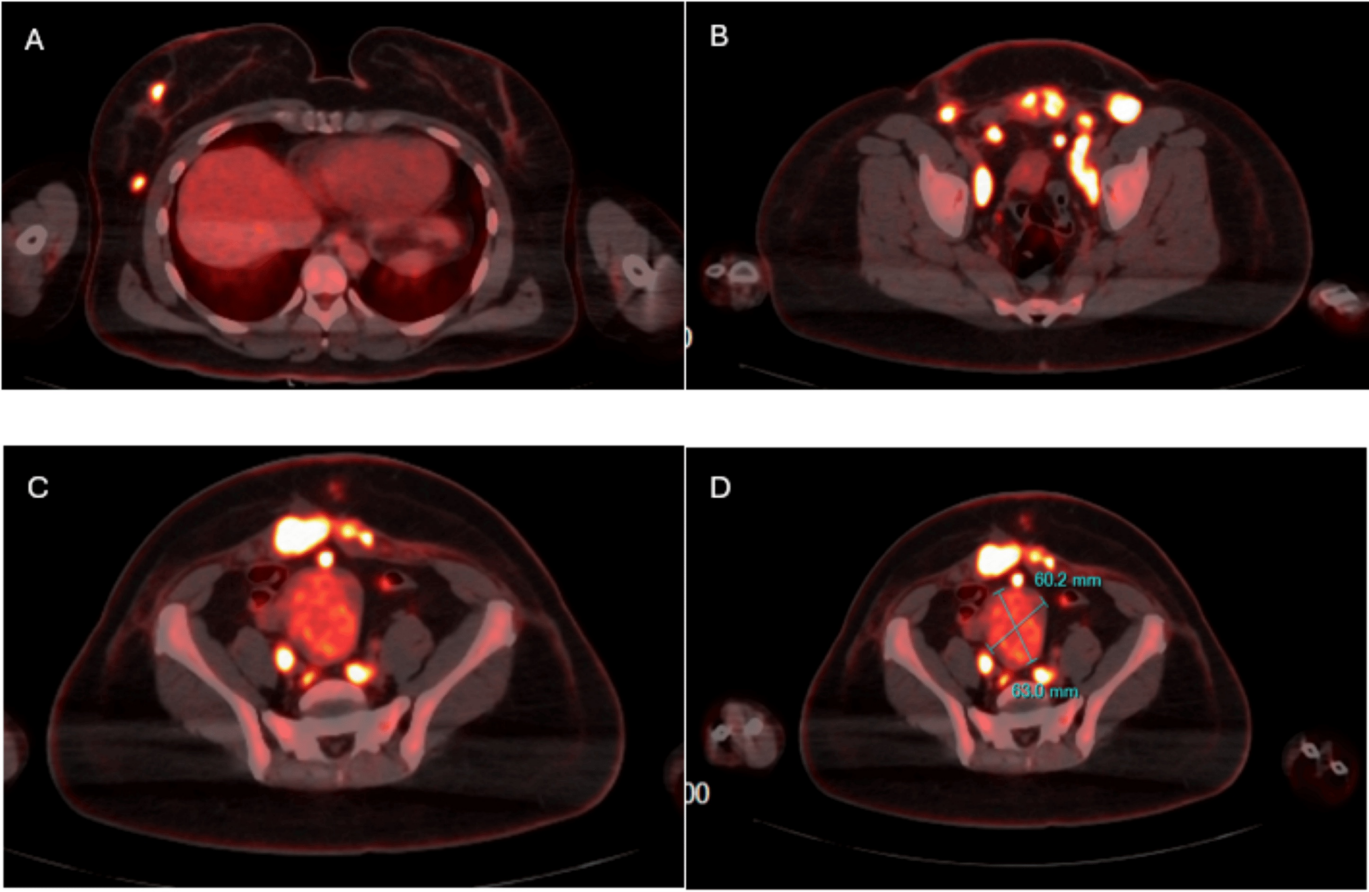
Whole body PET scan of patient. Whole body PET scan of the patient with the four parts (a)-(d) selected to show uptake in clinically relevant areas. (A) Above the diaphragm, radio-uptake in the right breast and axillary lymph node. (B) Below the diaphragm, the scan shows increased uptake in the pelvic lymph nodes visible in the pelvic section. (C) Below the diaphragm, the scan section focused on the uterus. There was radiotracer uptake within the uterus, along with evidence of omental metastasis. Increased uptake was also observed in the inguinal and pelvic lymph nodes, as well as in the omentum. (D) The same section from (C), but with measurements of the uterus listed. PET: positron emission tomography.

Magnetic resonance imaging (MRI) of the abdomen and pelvis revealed nodal masses measuring 24 × 28 mm on the right and 26 × 23 mm on the left (Figure 1). As part of the diagnostic workup, biopsies were obtained from the abdominal mass and the right inguinal lymph node, both confirming high-grade serous epithelial carcinoma. A positron emission tomography (PET) scan identified a solid uterine mass measuring 6.0 × 6.3 × 4.2 cm, with radiotracer uptake in the bilateral iliac, inguinal, and para-aortic lymph nodes, as well as omental metastases (Figure 2). Above the diaphragm, increased uptake was noted in the right breast and right axillary lymph nodes. Biopsy of the right breast revealed solid, nested, and micropapillary structures with marked nuclear atypia, pleomorphism, high nuclear-to-cytoplasmic ratios, prominent nucleoli, and brisk mitotic activity. Immunohistochemical staining demonstrated estrogen receptor and progesterone receptor negativity, HER2/neu overexpression, and Müllerian origin. The final diagnosis was HER2/neu-positive high-grade serous carcinoma of Müllerian origin. Following tumor excision, the patient's CA-125 levels decreased significantly to 35 U/mL.

She received Taxotere, carboplatin chemotherapy along with Herceptin/Perjeta (TCHP) for six cycles. She underwent surgical excision of the tumor mass by a surgical oncologist. 

## Discussion

It is relatively well-known that ovarian cancers can spread to breast tissue [1]. Metastases to the breast and axilla are rare, accounting for approximately 2% of all mammary malignancies [1]. Such metastases develop in the fifth or sixth decade. The most common reported sites of origin in the literature include lymphomas, skin (melanoma), lung, stomach, uterus, and ovary [2]. For ovarian cancer specifically, the most frequent ovarian carcinoma type to metastasize to the breast or axilla is high-grade serous carcinoma, with a frequency of 0.07% [3].

Although metastasis to the breast from non-breast primaries is uncommon, it may be under-recognized. Ovarian cancer that metastasizes to the breast can be challenging to distinguish from primary breast cancer due to similarities in both morphology and immunohistochemical profiles. These tumors can exhibit diverse histologic patterns, such as papillary, glandular, or solid structures, often accompanied by necrosis and high-grade cellular features [1]. In some cases, even if there is a known primary ovarian carcinoma, it may be a challenge to diagnose. Both breast and ovarian cancer can express ER and PR proteins, making their use insubstantial. PAX8 expression and WT1 are useful tumor markers that may aid in identifying ovarian origin. The former is a sophisticated tumor marker as nearly all ovarian tumors, excluding ovarian mucinous carcinomas they tend to be negative for PAX8 [4]. WT1 has demonstrated high sensitivity as a tumor marker for ovarian serous carcinoma, as it is strongly expressed in ovarian cancers while being rarely detected in breast cancers [5]. It should be noted that WT1 expression has some, albeit few, exceptions, as breast cancers with ductal or micropapillary components can express WT1. Therefore, immunohistochemical analysis of tumors is essential for accurate diagnosis, as there is some overlap.

Metastatic involvement of the breast and axilla requires prompt medical attention, as these cases are associated with a poor prognosis. In a study of 169 patients with metastatic tumors to the breast, the median survival from diagnosis was reported as 10 months, with survival times ranging from less than a month to 192 months [6]. Another study examining 85 patients found a median survival of 15 months, with a range spanning from under one month to 83 months [1]. The limited survival rates are largely attributed to the extensive tumor burden present at the time of diagnosis. 

To our knowledge, there are very few reports of HER2/neu-positive, high-grade ovarian serous carcinoma spreading to the breast or axilla. A study of 238 reports showed that, overall, HER2/neu-positive, high-grade, serous carcinoma is exceedingly rare [7]. Other reports have identified HER2/neu-positive mucinous carcinoma [8], high-grade serous adenocarcinoma [3], undifferentiated adenocarcinoma and moderately-differentiated adenocarcinoma [9], and serous cystadenocarcinoma [10]. All of which are varied in their histological features and tumor markers. Importantly, most cases of ovarian carcinoma spreading to the breast are triple-negative [7]. These distinctions are important as HER2/neu or PD-L1 status determines chemotherapy regimens and medical treatment.

It is important to diagnose malignancies accurately, as treatment options vary significantly. It is imperative to be efficient in these cases as patients with metastatic disease have a poor prognosis.

## Conclusions

This case illustrates a rare presentation of high-grade serous carcinoma of Müllerian origin with HER2/neu overexpression metastasizing to the breast and axillary lymph nodes. While HER2 positivity in this tumor type is uncommon, the case underlines the critical importance of thorough histopathological evaluation, immunohistochemistry, and clinical correlation in differentiating metastatic lesions from primary breast malignancies. Clear staging, detailed pathology reporting, and multidisciplinary coordination are essential for accurate diagnosis and appropriate management. Future reports should emphasize comprehensive histologic and molecular characterization to clarify diagnostic challenges and reinforce clinical relevance.
